# Validation and recalibration of sex estimation methods using pubic nonmetric traits for the Chilean population

**DOI:** 10.1007/s00414-024-03233-z

**Published:** 2024-04-13

**Authors:** Natalia Rojas González, Zuzana Obertová, Daniel Franklin

**Affiliations:** Centre for Forensic Anthropology, School of Social Sciences, The University of Western Australia, 35 Stirling HWY, Crawley, WA Australia

**Keywords:** Forensic anthropology, Sexual dimorphism, Sex estimation, Pelvic non-metric traits, Logistic regression, Chilean

## Abstract

Chile had a violent military coup (1973–1990) that resulted in 3,000 victims declared detained, missing or killed; many are still missing and unidentified. Currently, the Human Rights Unit of the Forensic Medical Service in Chile applies globally recognised forensic anthropological approaches, but many of these methods have not been validated in a Chilean sample. As current research has demonstrated population-specificity with extant methods, the present study aims to validate sex estimation methods in a Chilean population and thereafter establish population-specific equations. A sample of 265 os coxae of known age and sex of adult Chileans from the Santiago Subactual Osteology Collection were analysed. Visual assessment and scoring of the pelvic traits were performed in accordance with the Phenice (1969) and Klales et al. (2012) methods. The accuracy of Phenice (1969) in the Chilean sample was 96.98%, with a sex bias of 7.68%. Klales et al. (2012) achieved 87.17% accuracy with a sex bias of -15.39%. Although both methods showed acceptable classification accuracy, the associated sex bias values are unacceptable in forensic practice. Therefore, six univariate and eight multivariate predictive models were formulated for the Chilean population. The most accurate univariate model was the ventral arc at 96.6%, with a sex bias of 5.2%. Classification accuracy using all traits was 97.0%, with a sex bias of 7.7%. This study provides Chilean practitioners a population-specific morphoscopic standard with associated classification probabilities acceptable to accomplish legal admissibility requirements in human rights and criminal cases specific to the second half of the 20th century.

## Introduction

Chile was one of the many countries in Latin America immersed in a socio-political conflict between neoliberalism supporters and those who opposed them with socialist ideologies [1, 2]. This complex political situation contributed to the disruption of democracy in Chile through a violent military coup led by the armed forces in 1973. During 17 years of the dictatorial mandate, mass disappearances, illegal detentions, executions, and torture occurred, affecting 3,227 people, according to reports on human rights violations in Chile [1].

The latest Chilean public policy is the implementation of *‘The National Search Plan for Truth and Justice’*, which seeks to clarify the circumstances of victims’ disappearance and/or death and the continuation of the search, recovery and identification [3]. The Human Rights Unit (HRU) of the Chilean Forensic Medical Service is the only public institution in charge of analysing, selecting and sampling skeletal material to identify these victims [4]. Since 2007, following the recommendations of a panel of international forensic experts, positive identifications have been performed exclusively through DNA analysis conducted in accredited international laboratories [5]. However, the HRU employs a multidisciplinary approach, which includes anthropological analysis of all skeletal material [1].

Sex is an essential biological attribute when analysing unknown human remains in forensic investigations because accurate sex estimation has the possibility to eliminate individuals with inconsistent profiles from further investigative consideration in relation to identification (e.g., opposite sex) [6]. Skeletal sex estimation in adults relies on morphological and physiological differences between males and females [7]. This sexual dimorphism is determined by a complex interaction between genetic, functional and environmental factors [8, 9]. In forensic investigations, the areas in the pelvis are considered the most dimorphic and accurate for sex estimation in adults [10–12]. This is because the shape and functionality of the female pelvis are specific due to its obstetric adaptation, which, to some degree, transcends population variances [8, 13]. Morphoscopic (visual) and morphometric (metric) methods have been developed to assess sex using the pelvis. The former has been criticised for being subject to observer bias; however, practitioners still prefer visual rather than metric assessments because they are faster to apply, more cost-effective and straightforward [14, 15].

The most frequently used morphoscopic sex estimation method, also currently used by the HRU, is Phenice [16]. This standard was created using African-American and European-American individuals and involves observing three sexually dimorphic traits of pubic bone: ventral arc (VA); subpubic concavity (SPC); and the medial aspect of the ischio-pubic ramus (MA). The presence or absence of specific characteristics in those traits classifies the individual as female or male, respectively; the assignation of 2 of the 3 traits classifies sex. The reported original accuracy of this method is 96.00% (95.56% males; 96.84% females; -1.28% sex bias). Numerous validation studies of the original method in different population samples have been performed, resulting in accuracies ranging from 58.6 to 96.6% [12, 17–22]. The differences in achieved accuracy can be attributed to the application of the method to populations outside the original reference sample (e.g., distant geographically, genetically and/or temporally) as the level of sexual dimorphism is known to vary between populations [23, 24].

Klales et al. [22] revised the Phenice [16] method to facilitate the admissibility requirements in court (i.e., Daubert guidelines) by including an ordinal scale of scores and a regression analysis with classification probabilities [25]. Klales et al. [22] used the Hamann-Todd and the W.M. Bass skeletal collections of mixed ethnicity (predominantly from the U.S.) to develop their modified version of Phenice [16]. The accuracy of the logistic regression for estimating sex was 86.2% using all three traits (74.4% males; 98.0% females; -23.6% sex bias). The sex bias value in this method is massive, demonstrating a proportionately greater correct classification of one sex (female over male). Thus, the high differential bias renders the method’s practical application unreliable [26]. However, paradoxically, the authors never referred to this issue. The Klales et al. [22] method was also tested in non-US samples, showing correct classification accuracies ranging from 66 to 95% [27–30].

Considering the importance of providing scientific information in the medico-legal system, sex assignation should include statistical probability of correct classification established on population-specific data [26]. Thus, some researchers have recalibrated the logistic regression equation by Klales et al. [22] to include *population-specific* applications, all of which improved classification accuracy and decreased sex bias values [28–30].

A small number of studies have considered population-specific sex estimation standards for Chileans; all of them are morphometric, using measurements of long bones and the scapula [2, 8, 31]. However, forensic practitioners in the HRU still prioritise morphoscopic sex estimation methods, including Phenice [16]. Therefore, the present study is designed for direct end-user application and aims to examine the accuracy of the original Phenice [16] and Klales et al. [22] methods, and thereafter present new population-specific logistic regression equations for the Chilean population. The latter will be particularly important for those cases associated with the identification of human rights victims and unknown skeletal cases dated from the second half of the 20th century.

## Materials and methods

### Study sample

#### Human Rights Unit (HRU) skeletal collection

The modern skeletal collection from the HRU of the Chilean Forensic Medical Service comprises 110 individuals (77 male; 33 female) with years of death between 1990 and 1997. However, only 42 individuals (33 male; 9 females) between 28 and 89 years old had at least one *os coxa* to analyse. Thus, a randomly selected subsample of 22 individuals (13 males; 9 females) from this collection was used only for the intraobserver-agreement test.

#### Santiago Subactual Osteology (SSO) Collection

The skeletal collection from the University of Chile, ‘*Colección Osteológica Subactual de Santiago’*, translated as Santiago Subactual Osteology Collection, comprises 1,198 skeletonised individuals with known biological sex and age at death [32]. Those individuals were exhumed from the General Cemetery of Santiago under the Chilean Decree of Law 357, *General Regulation of Cemeteries*, Title IX *‘Distribution of corpses for the purpose of scientific investigation’* articles 79 and 80, which indicates that in circumstances where the burial term expired, cemeteries are allowed to donate the skeletal remains to medical schools and universities for scientific research purposes. Thus, age at death and sex are retrieved from the cemetery records. The sample comprises complete and incomplete skeletons, adults and subadults; therefore, all remains recorded as adults in the records were reviewed to select the sample for this study.

Sample selection included two main criteria: (i) only individuals aged ≥ 20 years old were selected, considering the statements by Phenice [16] that VA and SPC are not well developed in females before that age; and (ii) the individuals had at least one *os coxae* with the three pelvic traits able to be assessed. *Os coxae* with injuries (e.g., fractures) that affect normal morphology were excluded. The total sample examined comprised 265 individuals; 196 male and 69 female, between 20 and 96 years of age, with a date of death between 1950 and 1970 (Table 1). The left *os coxae* was selected for analysis, but the right side was used when the left was unavailable (in 55 cases). All the analyses of this study were developed using the SSO collection except for the intra-observer agreement test, for which the HRU collection was used (see above).

In consideration of the type of sample analysed (i.e., formal skeletal collections), the Human Ethics Office of the University of Western Australia has classified this study as exempt from ethics review (Ref code: 2021/ET000378).

**Table 1:** Age distribution of Chilean males and females in the SSO skeletal collection

Known age (years)	Male (n)	Female (n)	Total (n)
20–49	93	21	114
50–69	65	25	90
70+	38	23	61
Total	196	69	265

### Assessment and data collection

**Fig. 1:**
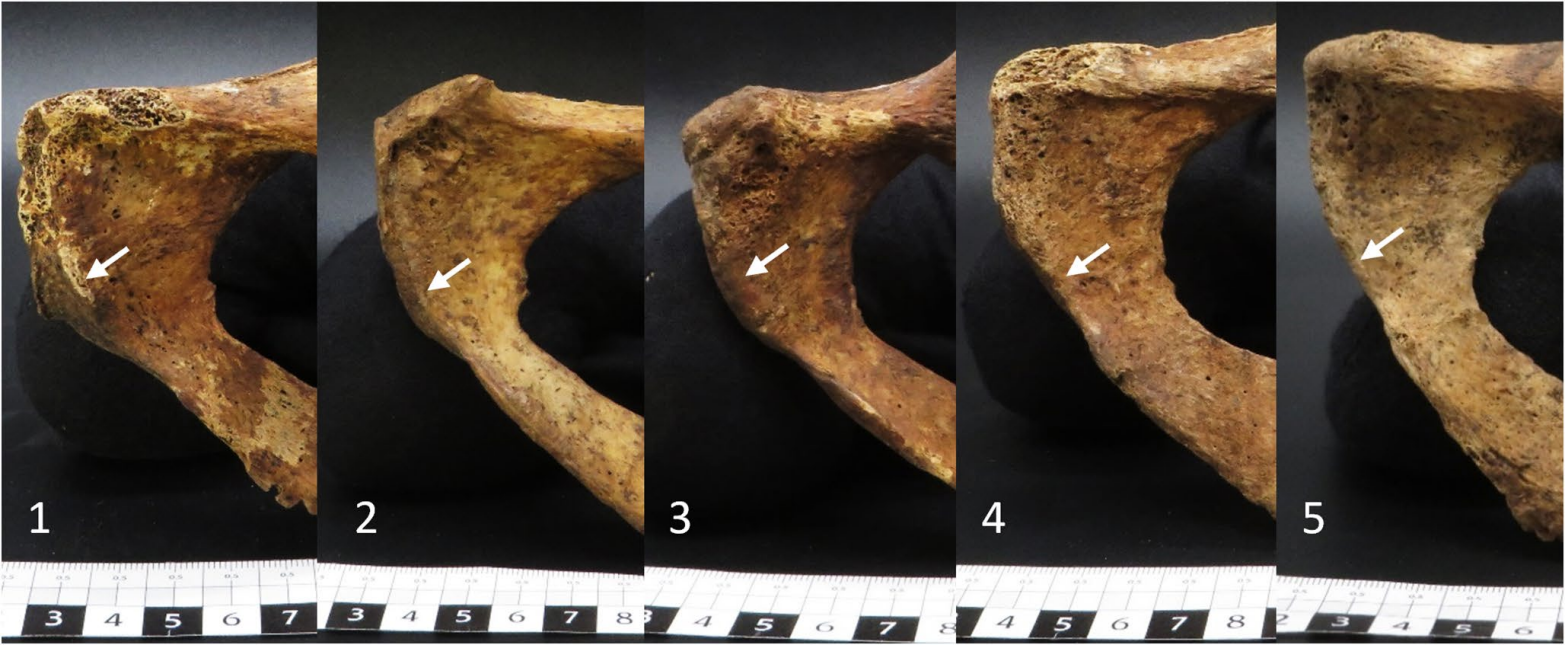
The ventral arc (white arrows) scores from 1 to 5, following Klales et al. [22], in the Chilean sample. Left innominate bones are orientated in ventral view, and all images are not scaled

The VA, SPC, and MA were visually evaluated and scored according to the illustrations and descriptions by Phenice [16] and Klales et al. [22] (e.g., Fig. 1). All assessments were performed with sex and age blinded to the observer (NRG). Each individual was classified as male or female according to the Phenice [16] criteria (i.e., 2/3 of the traits classify sex). Similarly, the scores of each pelvic trait were used in the logistic regression equation of Klales et al. [22]; values less than 0 are categorised as female, and greater than 0 are male. Following Press and Wilson [33] and as advised by Klales et al. [22], posterior probabilities for sex classification using the logistic regression score were calculated for each individual.

The study was performed first at the Chilean Forensic Medical Service (reliability test) and after at the University of Chile in Santiago, Chile (primary analyses). Access to both collections was granted upon request by the Institute Dr Carlos Ybar of the Chilean Forensic Medical Service and the Department of Anthropology of the University of Chile.

### Statistical analysis

All statistical analyses were conducted using *IBM SPSS Statistics version 29* and *Microsoft Excel* for *Microsoft 365 version 16.*

#### Intra-observer agreement

Before primary data collection, a precision test was performed (by NRG) to test the consistency of repeat assessments. A sample of 22 individuals aged 28 to 89 from the HRU skeletal collection was used to quantify the intra-observer agreement: 13 males (mean age = 48.8 years) and 9 females (mean age = 66.9 years). The specimens were analysed twice following Phenice [16] and Klales et al. [22]. The methods were assessed individually, without knowing the actual sex and age of the individuals. The analyses were performed with at least one week between re-assessment to reduce potential recall bias. Cohen’s Weighted Kappa test (*K*) was used to evaluate intra-observer error in scoring the three pelvic traits; these values were interpreted according to Landis and Koch [34].

#### Trait score distributions

Frequency distributions by sex using both methods were calculated separately. For Phenice [16], traits were scored as 1 for “present” and 0 for “absent”; for Klales et al. [22], scores 1 to 5 were cross-tabulated. In addition, a Chi-square test (Χ^2^) was applied to explore the association between sex and score frequency for each pelvic trait when applying both methods.

#### Validation of Phenice (1969) and Klales et al. (2012)

Sex assignation using both methods was compared with the recorded sex of each individual. The accuracy of the methods was analysed based on the percentage of correct classification and sex bias. The percentage of correct classification was calculated by comparing recorded and estimated sex; sex bias is the difference in classification accuracy between males and females, with a value of ≤ 5% deemed acceptable [35].

#### Population-specific models for the Chilean population

Univariate and multiple binary logistic regression (BLR) analyses were performed to derive Chile-specific sex estimation models for both methods.

Using the Phenice [16] method, sex was coded as 0 for males and 1 for females, following the method’s rule of trait ‘absent’ = male and trait ‘present’ = female. Classification accuracy according to individual and combined sex, and sex bias values were calculated. Thus, when applying the Chilean-specific logistic regression equations using this method, the individuals will be classified as male if the results are negative (< 0) and female if the results are positive (> 0).

In opposition, using the Klales et al. [22] method, sex was coded as 0 for females and 1 for males, following this method’s original rule indicating results less than zero are female and over zero are male. Classification accuracy according to individual and combined sex and sex bias values were calculated. Thus, when applying the Chilean-specific logistic regression equations using this method, all positive results will be classified as male (> 0), and the negative results will be classified as female (< 0). Therefore, based on the score, the probability of being female and male can be calculated following Press and Wilson [33] as advised by Klales et al. [22].

## Results

### Intra-observer agreement

The intra-observer reliability test showed Kappa values of > 0.90 for all pelvic traits using both methods, except for MA using Phenice [16], *K* = 0.82 (see Table 2).

**Table 2:** Intra-observer accordance for the Phenice [16] and Klales et al. [22] trait assessment

	Kappa value	
Pelvic Trait^a^	Phenice	Klales et al.
VA	0.91	0.91
SPC	1.00	0.92
MA	0.82	0.96

^a^ VA = ventral arc; SPC = subpubic concavity; MA = medial aspect of the ischiopubic ramus

### Frequency distributions

The frequency distribution of each trait for Phenice [16] and Klales et al. [22] are shown in Tables 3 and 4, respectively. Significant differences in score frequencies between females and males were observed for all pelvic traits for the Phenice [16], Χ^2^ (1, *N* = 265)  ≥ 143.36, *p* < 0.001, and Klales et al. [22] methods, Χ^2^ (4, *N* = 265)  ≥ 137.61, *p* < 0.001.

#### Ventral arc

Sixty-four of 69 females had a VA when assessed using Phenice [16]. In comparison, only four males displayed this feature when applying the same method (Table 3). For Klales et al. [22], a score of 1 was the most frequent for females (56.5%). Only one female scored 4, and none scored 5. Conversely, a score of 4 was the most frequent for males (52.6%), while scores of 1 and 2 were present in 2 male individuals each (Table 4).

**Table 3:** Pelvic trait assessment by sex using Phenice [16] standard to a Chilean population

		Trait assessment (%within recorded sex)	
Pelvic Trait^a^	Sex	Present	Absent
VA	Male	4 (2.0%)	192 (98.0%)
	Female	64 (92.8%)	5 (7.2%)
SPC	Male	4 (2.0%)	192 (98.0%)
	Female	47 (68.1%)	22 (31.9%)
MA	Male	5 (2.6%)	191 (97.4%)
	Female	53 (76.8%)	16 (23.2%)

^a^ VA = ventral arc; SPC = subpubic concavity; MA = medial aspect of the ischiopubic ramus

**Table 4:** Distribution of scores using the ordinal scale by Klales et al. [22] for each pelvic trait by sex to the Chilean sample

		Trait Score				
Pelvic Trait^a^	Sex	1	2	3	4	5
VA	Male	2 (1.0%)	2 (1.0%)	47 (24.0%)	103 (52.6%)	42 (21.4%)
	Female	39 (56.5%)	25 (36.2%)	4 (5.8%)	1 (1.4%)	0 (0.0%)
SPC	Male	0 (0.0%)	3 (1.5%)	94 (48.0%)	92 (46.9%)	7 (3.6%)
	Female	4 (5.8%)	43 (62.3%)	22 (31.9%)	0 (0.0%)	0 (0.0%)
MA	Male	0 (0.0%)	47 (24.0%)	88 (44.9%)	52 (26.5%)	9 (4.6%)
	Female	33 (47.8%)	29 (42.0%)	6 (8.7%)	1 (1.4%)	0 (0.0%)

^a^ VA = ventral arc; SPC = subpubic concavity; MA = medial aspect of the ischiopubic ramus

#### Subpubic concavity

Forty-seven of 69 females and only four males had a SPC when assessed following Phenice [16] (Table 3). For Klales et al. [22], over half of the female sample scored 2; no females scored 4 or 5. Nearly 95% of the male sample is divided between scores 3 and 4; no male individuals were assigned a score of 1 (Table 4).

#### Medial aspect of the Ischio-pubic ramus

Fifty-three of 69 females presented evidence of a ridge in the ischio-pubic ramus. In contrast, only five males showed the same feature when applying Phenice [16] (Table 3). For Klales et al. [22], 90% of the female sample is divided between scores 1 and 2; only one scored 4, and none scored 5. Close to half of the male sample scored 3, and no male individuals were assigned a score of 1 (Table 4).

### Classification accuracy of Phenice (1969) and Klales et al. (2012) in the Chilean population

Phenice [16], as applied to the Chilean sample, showed an overall classification accuracy of 96.98%, with a sex bias of 7.68% (see Table 5). The Klales et al. [22] method applied to the same sample achieved 87.2% accuracy, with a sex bias of -15.4% (see Table 5).

**Table 5:** Comparison between the accuracies of the original studies by Phenice [16] and Klales et al. [22] and the accuracies obtained with the Chilean population

	Phenice		Klales et al.	
	Chile	Original	Chile	Original
Male (*n* = 196)	98.98% (*n* = 194)	95.56%	83.2% (*n* = 163)	74.4%
Female (*n* = 69)	91.30% (*n* = 63)	96.84%	98.6% (*n* = 68)	98.0%
Overall (*n* = 265)	96.98% (*n* = 256)	96.00%	87.2% (*n* = 231)	86.2%
Sex bias	7.68%	-1.28%	-15.4%	-23.6%

### Population-specific predictive models for the Chilean population

Three univariate models (P1 to P3) and four multivariate models (PM1-PM4) were derived using the Phenice [16] scores (see Table 6). From the univariate models, the highest overall classification accuracy (96.6%) and the lowest sex bias (5.2%) were for Function P1 using the VA. The lowest overall classification accuracy (90.2%) and the highest sex bias (29.9%) were for Function P2 using the SPC (Table 6). Among the multivariate models, Function PM4, which incorporates all pelvic traits, had the highest overall classification accuracy at 97.0%, with a 7.7% sex bias, followed by Function PM1, using the combination of VA and SPC with 96.6% accuracy and a 5.2% sex bias. However, the latter equation showed that the SPC was not statistically significant (*p* > 0.05); thus, the accuracy is the same as the univariate equation P1 (only using VA). The least accurate function was PM3 (95.5%), using MA and SPC (Table 6).

**Table 6:** Chilean-specific functions, classification accuracies and sex bias derived by applying the Phenice [16] binomial scoring coded 0 as “absent” or 1 as “present”

		Accuracy (%)			
	Chilean-specific predictive models^a^	Male	Female	Total	Bias
	Univariate				
P1	6.42(VA) - 3.65	98.0	92.8	96.6	5.2
P2	4.63(SPC) - 2.17	98.0	68.1	90.2	29.9
P3	4.84(MA) - 2.48	97.4	76.8	92.1	20.6
	Multivariate				
PM1	5.58(VA) + 1.67(SPC) - 3.71*	98.0	92.8	96.6	5.2
PM2	5.57(VA) + 3.60(MA) - 4.27	98.0	92.8	96.6	5.2
PM3	5.49(MA) + 5.34(SPC) - 4.15	95.4	95.7	95.5	-0.3
PM4	3.90(VA) + 2.87(SPC) + 4.06(MA) - 4.57	99.0	91.3	97.0	7.7

^a^ VA = ventral arc; SPC = subpubic concavity; MA = medial aspect of the ischiopubic ramus

^*^SPC in this equation showed no statistical significance, *p* = 0.077

Sex coding: Female = 1; Male = 0

Three univariate (K1 to K3) and four multivariate models (KM1-KM4) were derived using scoring data following Klales et al. [22] (see Table 7). Among the univariate models, the highest overall classification accuracy (96.6%) and the lowest sex bias (5.2%) were for Function K1 using VA. The lowest overall classification (86.4%) and the highest sex bias (52.2%) were for Function K3 using the MA (Table 7). Among the multivariate models, Function KM1 using VA and SPC showed the highest classification accuracy (96.2%) and the lowest sex bias (4.6%). Function KM3, using the combination of traits MA and SPC, showed the lowest overall accuracy (94.0%) and the highest sex bias (17.3%) (Table 7).

**Table 7:** Chilean-specific functions, classification accuracies, and sex bias by applying the Klales et al. [22] ordinal scoring method

		Accuracy (%)			
	Chilean-specific predictive models^a^	Male	Female	Total	Bias
	Univariate				
K1	3.27(VA) - 7.89	98.0	92.8	96.6	5.2
K2	4.26(SPC) - 11.30	98.5	68.1	90.6	30.4
K3	2.66(MA) - 5.09	100.0	47.8	86.4	52.2
	Multivariate				
KM1	2.39(VA) + 2.57(SPC) - 13.16	97.4	92.8	96.2	4.6
KM2	2.66(VA) + 1.17(MA) - 9.18	98.0	91.3	96.2	6.7
KM3	2.33(MA) + 4.31(SPC) - 16.59	98.5	81.2	94.0	17.3
KM4	2.00(VA) + 2.58(SPC) + 1.12(MA) - 14.67	97.4	89.9	95.5	7.5

^a^ VA = ventral arc; SPC = subpubic concavity; MA = medial aspect of the ischiopubic ramus

Sex coding: Female = 0; Male = 1

## Discussion

The present study assessed the performance of two well-known morphoscopic sex estimation methods in a Chilean population. Currently, there are no validation studies for those methods specific to the Chilean population. Therefore, the results of this study serve to facilitate informing forensic practitioners of error rates associated with both methods. The classification accuracies obtained were over 85%. However, they demonstrated a high level of misclassification between sexes, revealing the need for population-specific models. Therefore, 14 population-specific equations derived from Chilean data were presented, most providing correct classification according to sex > 90% and half with an associated sex bias value of ~ 5%. These functions will enhance the ability of forensic practitioners working with Chilean human rights cases and unknown skeletal remains associated with atrocities of the second half of the 20th century to achieve more accurate outcomes leading to potential identifications.

### Intra-observer agreement

The reliability of any forensic method (i.e., quantification of observer’s error) is just as important as achieving an accurate classification of sex; ethical and professional practice mandates that you cannot have one without the other. According to the Kappa statistic values presented here, all pelvic traits for both methods showed an ‘almost perfect agreement’ (*K* > 0.81), according to Landis and Koch [34]. The only trait that showed a Kappa value under 0.90 was the MA when applying the Phenice [16] method. This result corresponds with a comparable study testing the same method in a Portuguese population, indicating that MA was the least reliable trait among the three assessed [21]. In addition, this result aligns with the warnings by Phenice [16], who noted that the medial aspect of the ischiopubic ramus was likely to be the most ambiguous trait of the three assessed.

### Frequency distributions

When analysing the distribution of the presence-absence of features applying Phenice [16], the most accurate in females was the VA, with only five individuals misclassified, and the least accurate was the SPC. The number of misclassifications in males was noticeably low for all traits (< 3.0%). VA and SPC showed the highest accuracies in males, and MA was the lowest. Overall, for both sexes, the VA was shown to be the most accurate sex indicator, which accords with Phenice [16] and previous studies examining this method [19, 36, 37]. On the other hand, similarly to this study, the MA has also been reported as the least accurate indicator in European males [18], Mexicans [28], Hispanics [29] and Portuguese [21].

When analysing the frequency distribution after applying the Klales et al. [22] scoring system, females predominantly clustered into the lower scores (1 and 2), with a score of 5 not being assigned. Similar score distributions were described by Gómez-Valdés et al. [28] in Mexican females. A score of 3 was present in less than 10.0% of Chilean females for each trait, except for the SPC. Most females scored 2 (62.3%) and 3 (31.9%) for the SPC, indicating predominately intermediate shapes in this trait for Chilean females. Further, 48.0% of males also scored 3 in this trait, showing a considerable overlap between sexes, which could indicate a smaller level of sexual dimorphism for this feature in this population.

Males were slightly more variable than females in score frequency when applying Klales et al. [22], similar to what was observed in the *‘Hispanic’* samples in the study by Klales and Cole [29]. Chilean males were mainly grouped into mid-high scores (3 and 4), similar to the scores reported by Kenyhercz et al. [30] for their *‘Hispanic’* sample. ‘*Hispanic’* has been defined by the U.S. Census Bureau as a *‘person of Cuban, Mexican, Puerto Rican, South or Central American, or other Spanish culture or origin, regardless of race’* [38]. Thus, it is not surprising that these scores are similar to those recorded by previous studies on Hispanic populations, considering that the term ‘*Hispanic’* encompasses all people of Spanish lineage [39]. Only two Chilean males scored 1, and less than 5.0% of the male sample for each trait scored 5, except for the VA. These results indicate that Chilean males predominantly display intermediate shapes in most pelvic traits and are less robust overall than the males in the sample analysed by Klales et al. [22].

### Classification accuracy of Phenice (1969) and Klales et al. (2012)

The Phenice [16] method applied to the Chilean population performed as expected, with 96.98% correct classification, slightly higher than reported in the original study (Table 5). This result is comparable to Rae Jager and Eliopoulos [21], who examined a Portuguese population, achieving 96.0% accuracy. Similarities in skeletal morphology might exist between these populations, considering that Spain conquered Chile in 1541, and the immigration of other European countries (including Portugal) to Chile started at the beginning of the 19th century [40].

The sex bias value for the Chilean population is higher than reported by Phenice [16] (Table 5). Only two males (65 and 78 years old) and six females were misclassified; all females of those misclassified were over 50 years old (average age 63 years old). Previous studies suggested that the accuracy of the Phenice [16] method decreases in females with increasing age at death, which corresponds with the results of this study [12, 17].

A recent study by DesMarais et al. [41] examined age relative to greater sciatic notch (GSN) morphology in Australian females; it was demonstrated that this trait becomes narrower with increasing age. Interestingly, the latter was significant only in menopausal females (> 49 years old) and not in males of the same age. This finding could indicate that female pelvic morphology changes as age increases, affecting the GSN and potentially other features in the pelvis. However, it is worth noting that Sharma et al. [42] examined morphological changes in pelvic bone remodelling in women through life, with specific reference to parturition. That study concluded that the phenotypic plasticity detected in older women was due to childbirth and not related to increasing age, as other studies suggested. The present study has no clinical information about parturition, so the hypothesis of Sharma et al. [42] cannot be tested. Nevertheless, all females misclassified in this study were > 50 years old, with 50 years being the average menopause age in Chile [43].

The overall original classification accuracy of Klales et al. [22] was shown to be similar to that achieved in the present study (87.2%) (Table 5). However, the percentage of correct classification achieved in this study was lower than in other non-U.S. populations testing the same method, such as Mexico (95%) [28], South Africa (93.5%) [30], and Portugal (92.7%). A possible explanation for this result is that due to the variations in levels of sexual dimorphism between populations, the range of variation and descriptions given by Klales et al. [22] (scoring 1 to 5) might not align with the degree of morphological variation existent in the Chilean population.

Relative to the sex bias values, the Klales et al. [22] method showed a lower value in this population than in the original study (Table 5). However, it was still unacceptably high at -15.4%. From a total of 196 males, 33 aged 20 to 94 (49 years old average age) were misclassified; no evident trend in the age distribution of these individuals was observed. The fact that a higher percentage of males was classified as females could indicate that Chileans have a smaller degree of sexual dimorphism than the population analysed by Klales et al. [22] and/or the range of variation proposed by Klales et al. [22] does not fit with the morphology of Chilean males.

Furthermore, the 33 males misclassified using Klales et al. [22] included the same two males misclassified using the Phenice [16] method (see above). Further, the single female misclassified using Klales et al. [22] was similarly misclassified using the Phenice [16] method. Thus, those three individuals are likely outliers relative to sex in the Chilean sample, especially considering they were misclassified using both standards. It is also possible that biological sex is incorrectly recorded in the collection records.

### Population-specific models

A total of 14 functions were formulated using the Chilean population data. The univariate population-specific equations using VA (P1) and the multivariate equation using the combination of all three pelvic traits (PM4) showed higher overall accuracies than the original method of Phenice, both functions with a sex bias slightly over the acceptable limit (5.2% and 7.7%). The univariate population-specific equation using VA (K1) and the multivariate equation using the combination of VA and SPC (KM1) showed better overall accuracies than the original Klales’s method, with a sex bias of 5.2% and 4.6%, respectively. These results support previous studies indicating that population-specific equations outperform the original non-specific methods, increasing percentage of correct classification and reducing the sex bias [28–30, 44].

The VA was the most accurate trait in the univariate functions for the Chilean population. This supports Phenice’s statement, indicating this feature *‘is the least likely to be ambiguous’* [16]. In addition, this also accords with previous studies indicating that the VA is the most accurate indicator of sex [19, 21, 28, 29]. Multivariate functions varied in classification performance; from the eight proposed, the most accurate included all pelvic traits (PM4) using Phenice’s method, and the function that includes the VA and SPC (KM1) using Klales et al. [22], both with over 96.0% overall correct classification.

When comparing univariate and multivariate functions, the univariate function analysing the VA is the most accurate, considering the percentage of correct classification and the sex bias value. Using this univariate function will be beneficial in analysing human rights and forensic cases, especially because most associated skeletal remains are found incomplete or fragmented.

Finally, although the focus of this study was to create population-specific models to be applied mainly in cases of human rights, and to some extent to criminal cases of the same temporality (~ 1970s), it would be beneficial to explore if these models could be applied with the same accuracy to contemporary forensic cases, or if there is a need to update these models to the modern contemporary population.

### Limitations of the study

The main limitation of this study concerns ‘*collection biases*’ inherent to the analysis of physical skeletal collections. These biases can include the under-representativeness of one particular sex, socio-economic status, and age distribution (amongst other factors) [24, 45]. The present study has an under-representation of females, representing only 26.0% of the total sample. 53.6% of the female sample is between 50 and 79 years old, with the male sample more equally distributed relative to age. In addition, most individuals analysed came from areas of low-income status, occupying the cheapest burial sites in the General Cemetery of Santiago [32]. It is acknowledged that the equations derived from the data analysed are optimised for the sample studied [23, 24]. Therefore, applying these models to a broader, more diverse Chilean sample (e.g., including different socio-economic backgrounds) needs to be tested as adjustments could be needed.

## Conclusion

The present study aimed to evaluate the performance of the Phenice [16] and Klales et al. [22] methods in a skeletal sample representative of the Chilean population. Both standards showed acceptable correct classification accuracies (> 85%); however, the Phenice [16] method performed more accurately in this population relative to correct classification (96.98%) and sex bias (7.68%) values. Nevertheless, both standards exposed unacceptable levels of sex bias (i.e., absolute value over 5%) that could lead to errors in the estimations, specifically misclassifying one sex relative to the other. Thus, these results demonstrated the need for population-specific models to ensure high classification accuracy and lower sex bias values to reduce potential misidentifications. Population-specific functions were shown to increase classification accuracy and reduce sex bias values. The application of those models will help Chilean forensic practitioners undertake a more accurate assessment of referred skeletal remains associated with violations against human rights in that country and unknown skeletal cases dated from the second half of the 20th century.

## Data Availability

Access to the skeletal collections and associated demographic data used in this study can be made through a request sent to the Department of Anthropology of the University of Chile and the Dr Carlos Ybar Institute of the Chilean Forensic Medical Service. Data related to this study is housed within the University of Western Australia and can be made by request, according to their guidelines.

## References

[1] Garrido Varas C, Intriago Leiva M (2012) The Unidad Especial De Identificación Forense and Human rights in Chile. Cadernos De GEEvH 1:32–41

[2] Ross AH, Manneschi MJ (2011) New Identification Criteria for the Chilean Population: estimation of sex and stature. Forensic Sci Int 204:206. e201-206.e203 10.1016/j.forsciint.2010.07.028 20728293

[3] Gobierno de Chile (2023) Presidente Boric Lanza Plan Nacional De Búsqueda De Víctimas. de Desaparición Forzada en Dictadura

[4] Herrera MJ, Retamal R (2017) Reliability of Age Estimation from Iliac Auricular Surface in a subactual Chilean sample. Forensic Sci Int 275:317. e1-317.e4. 10.1016/j.forsciint.2017.01.029 10.1016/j.forsciint.2017.01.029 28314517

[5] Intriago Leiva M, Uribe Tamblay V, Garrido Varas C (2020) The Chilean experience in forensic identification of human remains. In: Parra RC, Zapico SC, Ubelaker DH (eds) Forensic Science and Humanitarian Action: interacting with the Dead and the living, 1st edn. John Wiley & Sons Ltd, pp 703–714. 10.1002/9781119482062.ch46

[6] Swift L, Obertova Z, Flavel A, Murray K, Franklin D (2023) Estimation of sex from cranial measurements in an Australian Population. Australian J Forensic Sci 55:755–777. 10.1080/00450618.2022.2081358 10.1080/00450618.2022.2081358

[7] Christensen AM, Passalacqua NV, Bartelink EJ (2019) Sex estimation. Forensic Anthropology: current methods and practice. Elsevier Science & Technology, San Diego, pp 243–270

[8] Carvallo D, Retamal R (2020) Sex estimation using the proximal end of the femur on a modern Chilean sample. Forensic Sci International: Rep 2:100077. 10.1016/j.fsir.2020.100077 10.1016/j.fsir.2020.100077

[9] Garvin HM, Klales AR (2020) Adult skeletal sex estimation and global standardization. Forensic Sci Humanitarian Action 199–209. 10.1002/9781119482062.ch14

[10] Black V (2021) Sex estimation using geometric morphometrics: evaluation of elements of the Pubis. Forensic Anthropol 4:47–56. 10.5744/fa.2020.0019 10.5744/fa.2020.0019

[11] Walker PL (2005) Greater sciatic notch morphology: sex, Age, and Population differences. Am J Phys Anthropol 127:385–391. 10.1002/ajpa.10422 15693026 10.1002/ajpa.10422

[12] Ubelaker DH, Volk CG (2002) A test of the Phenice Method for the estimation of sex. J Forensic Sci 47:19–24. 10.1520/JFS15200J 12064650 10.1520/JFS15200J

[13] Franklin D, Marks MK (2022) The Professional Practice of Forensic Anthropology: contemporary developments and cross-disciplinary applications. Wiley Interdisciplinary Reviews: Forensic Sci 4:e1442. 10.1002/wfs2.1442 10.1002/wfs2.1442

[14] Avent PR, Hughes CE, Garvin HM (2021) Applying posterior probability informed thresholds to traditional cranial trait sex estimation methods. J Forensic Sci. 10.1111/1556-4029.14947 34799862 10.1111/1556-4029.14947

[15] Klales AR (2020) Practitioner Preferences for Sex Estimation from Human Skeletal Remains Sex Estimation of the Human Skeleton: History, Methods, and Emerging Techniques Academic Press. pp. 11–24. 10.1016/B978-0-12-815767-1.00006-7

[16] Phenice TW (1969) A newly developed visual method of sexing the Os pubis. Am J Phys Anthropol 30:297–301. 10.1002/ajpa.1330300214 5772048 10.1002/ajpa.1330300214

[17] Lovell NC (1989) Test of Phenice’s technique for determining sex from the Os pubis. Am J Phys Anthropol 79:117–120. 10.1002/ajpa.1330790112 2750876 10.1002/ajpa.1330790112

[18] MacLaughlin SM, Bruce MF (1990) The accuracy of sex identification in European skeletal remains using the phenice criteria. J Forensic Sci 35:1384–1392. 10.1520/JFS12974J 2262774 10.1520/JFS12974J

[19] Johnstone-Belford E, Flavel A, Franklin D (2018) Morphoscopic observations in clinical pelvic MDCT scans: assessing the Accuracy of the phenice traits for sex estimation in a Western Australian Population. J Forensic Radiol Imaging 12:5–10. 10.1016/j.jofri.2018.02.003 10.1016/j.jofri.2018.02.003

[20] Oghenemavwe LE, Oludiniwa AN (2022) Morphoscopic approaches for Age and Sex Estimation of Unprofiled Hip bones: a study of Hip Bone Collections of Department of Anatomy Museum University of Port Harcourt, Nigeria. J Morphological Sci 39:132–139. 10.51929/jms.39.132.2022 10.51929/jms.39.132.2022

[21] Rae Jager V, Eliopoulos C (2023) Sex Assessment from the Pelvis: a Test of The Phenice (1969) and Klales (2012) Methods. Forensic Science, Medicine, and Pathology. 10.1007/s12024-023-00685-4 10.1007/s12024-023-00685-4 PMC11525382 37535230

[22] Klales AR, Ousley SD, Vollner JM (2012) A revised method of sexing the Human Innominate using Phenice’s nonmetric traits and statistical methods. Am J Phys Anthropol 149:104–114. 10.1002/ajpa.22102 22714398 10.1002/ajpa.22102

[23] Cattaneo C, Mazzarelli D, Cappella A et al (2018) A modern documented Italian identified skeletal Collection of 2127 skeletons: the CAL Milano Cemetery skeletal Collection. Forensic Sci Int 287. 10.1016/j.forsciint.2018.03.041. :219.e211-219.e215 10.1016/j.forsciint.2018.03.041 29703624

[24] Franklin D, Blau S (2020) Physical and Virtual Sources of Biological Data in Forensic Anthropology: Considerations Relative to Practitioner and/or Judicial Requirements. In: Obertová Z, Stewart A, Cattaneo C, eds. Statistics and Probability in Forensic Anthropology Academic Press, London, UK. pp. 17–45. 10.1016/B978-0-12-815764-0.00008-3

[25] Grivas CR, Komar DA (2008) Kumho, Daubert, and the Nature of Scientific Inquiry: implications for forensic Anthropology. J Forensic Sci 53:771–776. 10.1111/j.1556-4029.2008.00771.x 18489550 10.1111/j.1556-4029.2008.00771.x

[26] Franklin D (2023) Estimation of Skeletal Sex. In: Houck MM, ed. Encyclopedia of Forensic Sciences, Third Edition Elsevier, Oxford. pp. 292–303. 10.1016/B978-0-12-823677-2.00098-2

[27] Toon C, Garcia de Leon J (2014) A Comparison of the Klales (2012) and Phenice (1969) methods of Sex Estimation on a Modern Colombian sample. Poster presentation in the 66th Annual Scientific Meeting of the American Academy of Forensic Science, Seattle, Washington

[28] Gómez-Valdés JA, Menéndez Garmendia A, García‐Barzola L et al (2017) Recalibration of the Klales et al.(2012) method of sexing the human innominate for Mexican populations. Am J Phys Anthropol 162:600–604. 10.1002/ajpa.23157 28117482 10.1002/ajpa.23157

[29] Klales AR, Cole SJ (2017) Improving nonmetric sex classification for hispanic individuals. J Forensic Sci 62:975–980. 10.1111/1556-4029.13391 28070893 10.1111/1556-4029.13391

[30] Kenyhercz MW, Klales AR, Stull KE, McCormick KA, Cole SJ (2017) Worldwide Population Variation in Pelvic sexual Dimorphism: A validation and Recalibration of the Klales method. Forensic science international 277:259. e251-259. e258. 10.1016/j.forsciint.2017.05.001 10.1016/j.forsciint.2017.05.001 28666560

[31] Garrido-Varas C, Thompson T, Campbell A (2014) Parámetros Métricos para la Determinación de sexo en restos Esqueletales Chilenos Modernos. Chungará (Arica) 46:285–294. 10.4067/S0717-73562014000200009 10.4067/S0717-73562014000200009

[32] Meza-Escobar O, Galimany J, González-Oyarce R, Barreaux Höpfl N (2023) The Colección Osteológica Subactual De Santiago: origin and current state of a documented skeletal Collection from Chile, Latin America. Forensic Sci 3:80–93. 10.3390/forensicsci3010008 10.3390/forensicsci3010008

[33] Press SJ, Wilson S (1978) Choosing between logistic regression and Discriminant Analysis. J Am Stat Assoc 73:699–705. 10.2307/2286261 10.2307/2286261

[34] Landis JR, Koch GG (1977) The measurement of Observer Agreement for Categorical Data. Biometrics 33:159–174. 10.2307/2529310 843571 10.2307/2529310

[35] Franklin D, Cardini A, Flavel A, Marks M (2014) Morphometric analysis of pelvic sexual dimorphism in a Contemporary Western Australian Population. Int J Legal Med 128:861–872. 10.1007/s00414-014-0999-8 24789357 10.1007/s00414-014-0999-8

[36] Sutherland LD, Suchey JM (1991) Use of the ventral Arc in Pubic Sex determination. J Forensic Sci 36:501–511. 10.1520/jfs13051j 2066725 10.1520/jfs13051j

[37] Rogers T, Saunders S (1994) Accuracy of sex determination using morphological traits of the human pelvis. J Forensic Sci 39:1047–1056. 10.1520/jfs13683j 8064263 10.1520/jfs13683j

[38] United States Census Bureau (2022) About the Hispanic Population and its origin. United States Government

[39] Flores-Hughes G (2006) The origin of the term hispanic. Harv J Hispanic Policy 18:81–84

[40] Gutiérrez H (1989) La Inmigración Española, Italiana y Portuguesa: Chile 1860–1930. United Nations Economic Commission for Latin America and the Caribbean. pp. 61–79

[41] DesMarais A, Obertova Z, Franklin D (2023) The influence of age on Greater sciatic notch morphology: testing the Walker Method in an Australian Population. Int J Legal Med. 10.1007/s00414-023-02988-1 37055626 10.1007/s00414-023-02988-1 PMC10772010

[42] Sharma K, Gupta P, Shandilya S (2016) Age related changes in Pelvis size among adolescent and adult females with reference to parturition from Naraingarh, Haryana (India). HOMO 67:273–293. 10.1016/j.jchb.2016.04.002 27157866 10.1016/j.jchb.2016.04.002

[43] Instituto Nacional de Estadísticas (2018) Síntesis de Resultados, Censo 2017. Instituto Nacional de Estadísticas (INE), Chile pp. 27

[44] Lye R, Obertová Z, Bachtiar NA, Franklin D (2024) Validating the use of clinical MSCT scans for cranial nonmetric sex estimation in a Contemporary Indonesian Population. Int J Legal Med. 10.1007/s00414-024-03176-5 38300302 10.1007/s00414-024-03176-5 PMC11164787

[45] Henderson CY (2018) Introduction. In: Henderson CY, Alves Cardoso F (eds) Identified skeletal collections: the Testing Ground of Anthropology? Archaeopress Publishing Ltd., Oxford, UK, pp 1–8

